# Phonemes: Lexical access and beyond

**DOI:** 10.3758/s13423-017-1362-0

**Published:** 2017-09-05

**Authors:** Nina Kazanina, Jeffrey S. Bowers, William Idsardi

**Affiliations:** 1 School of Experimental Psychology, University of Bristol, 12a Priory Road, Bristol, BS8 1TU UK; 2 Department of Linguistics, University of Maryland, 1401 Marie Mount Hall, College Park, MD 20742 USA

**Keywords:** Access codes to lexicon, Lexical access, Lexical representation, Phonemes, Phonological form, Speech perception, Speech segmentation, Units of speech perception

## Abstract

Phonemes play a central role in traditional theories as units of speech perception and access codes to lexical representations. Phonemes have two essential properties: they are ‘segment-sized’ (the size of a consonant or vowel) and abstract (a single phoneme may be have different acoustic realisations). Nevertheless, there is a long history of challenging the phoneme hypothesis, with some theorists arguing for differently sized phonological units (e.g. features or syllables) and others rejecting abstract codes in favour of representations that encode detailed acoustic properties of the stimulus. The phoneme hypothesis is the minority view today. We defend the phoneme hypothesis in two complementary ways. First, we show that rejection of phonemes is based on a flawed interpretation of empirical findings. For example, it is commonly argued that the failure to find acoustic invariances for phonemes rules out phonemes. However, the lack of invariance is only a problem on the assumption that speech perception is a bottom-up process. If learned sublexical codes are modified by top-down constraints (which they are), then this argument loses all force. Second, we provide strong positive evidence for phonemes on the basis of linguistic data. Almost all findings that are taken (incorrectly) as evidence against phonemes are based on psycholinguistic studies of single words. However, phonemes were first introduced in linguistics, and the best evidence for phonemes comes from linguistic analyses of *complex* word forms and sentences. In short, the rejection of phonemes is based on a false analysis and a too-narrow consideration of the relevant data.

Within traditional linguistic theory, phonemes are units used to represent the ‘the psychological equivalent of a speech sound’ (Baudouin de Courtenay, 1972, p. 152,) or the psychophonetic or ideal sound forms of words also known as ‘phonological forms’ (Sapir, 1921, p. 55). Phonemes play a central role in explaining a large range of linguistic phenomena, from historical changes in pronunciation of words to dialectal variation to children’s speech or to how morphemes or words change when they combine into a larger sequence.

From a wider perspective that includes speech processing, the traditional view ascribes to phonemes two additional properties. On the speech production side, phoneme-based phonological representations should be translatable into a set of articulatory-motor control processes (Guenther, 2016). On the speech perception side, phonemes should be extractable from an acoustic signal and serve as access codes to words (i.e. it should be possible to map an acoustic signal to a sequence of phonemes in order to access lexical representations in long-term memory). This latter idea has been challenged by speech-perception theorists who claim that there are no acoustic invariances that characterize phonemes across contexts that allow speech stream to be parsed into phonemes (A. M. Liberman 1996), and by researchers who fail to obtain empirical evidence for phonemes (Perkell & Klatt 1986). Indeed, many theories and models of spoken word identification eschew phonemes in favour of alternative sublexical access codes, for example, position-specific allophones or (demi-) syllables.

In this article we consider conceptual and empirical challenges to the phoneme. One common feature of these criticisms is that they are predominantly advanced in the context of theories addressing monomorphemic single-word identification. Yet a key consideration for units of lexical representation is that they should be able to support linguistic computations across *all* levels of linguistic processing (A. M. Liberman, 1998). Indeed, the listener’s ultimate goal is not to identify sublexical units or single words but to understand the meaning of any one of a boundless number of novel phrases and sentences (Hickok & Poeppel, 2007; Pisoni & Luce, 1987). This involves recognising derived or inflected forms of words and establishing and interpreting grammatical relations between words or phrases. Even a simple phrase such as *John*’*s dog* requires establishing a relation between the possessive *John*’*s* (constructed by the syntax and not stored in the lexicon) and the base *John* (stored in the lexicon). The access codes to words need to support transparency of relations like this. Thus, we reconsider the claims made in the context of single words (Part 2) and pay special attention to arguments in favour of phonemes derived from linguistic analysis of more complex items (Part 3). It is the linguistic arguments that provide the strongest evidence for the psychological reality of phonemes as access units in speech perception that can support further language comprehension.

The organisation of the article is as follows. Part 1 defines the phoneme from the perspective of linguistic theory and discusses which properties it must have in order to enable an interface between lexical representation and their acoustic and articulatory-motor counterparts. Part 2 discusses conceptual and empirical challenges to the claim that phonemes serve as sublexical access codes to phonological word forms. On alternative views, the sublexical units are items other than phonemes or phonemes are artefacts of an alphabetical reading system. In each case, we show that the rejection of phonemes as a general feature of speech perception is unjustified. Part 3 provides a set of arguments for indispensability of the phoneme from various linguistic phenomena, ranging from single words to phrases. Indeed, phonemes were first proposed out of linguistic considerations, and linguistic evidence continues to provide the best evidence for their existence. Part 4 discusses a way of including phonemes into models of speech processing.

## Part 1: Defining the phonemic code

A considerable share of the speaker’s linguistic knowledge is knowledge about words. An average speaker retains knowledge of tens of thousands of distinct word forms that enable reference to a wide range of objects, properties and events. Most generally, knowing a word amounts to knowing the link between a sound form (aka ‘phonological form’) and a meaning, as well as morphosyntactic properties of the word, such as grammatical category, gender, and so forth. Words (aka lexical entries) are stored in the *lexicon*, a long-term memory repository for words and significant subword parts (morphemes).

Understanding how phonological forms of words are stored in the lexicon is key for any theory of language. The boundary conditions are that a language user should be able to recognise the phonological forms of words during speech comprehension and utter them appropriately in language production. A traditional answer from linguistic theory (Dresher, 2011; Jones, 1950; Sapir, 1921) is that words are represented in long-term memory as sequences of *phonemes*, that is, abstract and discrete symbolic units of a size of an individual speech segment, such as a consonants or vowel (yet not identical to them). A phonological form of a word is an *ordered sequence of phonemes*, for example, the sequence of phonemes /k/ - /æ/ - /t/ (more succinctly, /kæt/) refers to a meowing domesticated feline animal or /d∧k/ to a quacking avian. Apart from special cases such as homonymy or polysemy, two words that are distinct in meaning differ in phonological form, with a minimal difference being exactly one phoneme within the same position in the word (e.g. /kæt/ ‘cat’ vs. /mæt/ ‘mat’). Furthermore, different words can employ the same set of phonemes but in different orders (e.g. *cat * /kæt/ vs. *act * /ækt/ vs. *tack * /tæk/). A language typically uses a repertoire of a few dozens of phonemes that are combined to produce all of the thousands of word forms.

An essential property of the phoneme is that it is abstract. Individual instances of consonants and vowels are not phonemes as such, but rather an articulatory or acoustic realisation of a phoneme. The claim that phonemes are ‘segment-sized’ thus reflects the idea that each phoneme maps to a consonant or vowel segment (i.e. ‘phone’) when the phonemic representation is uttered (although, in some cases this mapping may be obscured by phonological processes; Chomsky & Halle, 1968). That phonemes are more abstract than phones is evident by comparing forms such as /kæt/ ‘cat’ and /d∧k/ ‘duck’, which both contain the phoneme /k/ even though it is realised as two different phones—an aspirated [k^h^] in *cat* and a plain or unreleased [k˺] in *duck*. This exemplifies a more general point: phonemes may be realised via different phones depending on the position within the syllable or word, on the neighbouring sounds, on whether the phoneme occurs within a stressed or unstressed syllable, and other factors. So, the American English phoneme /t/ is realized as an aspirated [t^h^] syllable-initially as in *top*, as an unaspirated [t] following /s/ as in *star*, or as an unreleased [t˺] in the syllable-final position as in *cat*. The above statement is an instance of a *phonological rule* of American English whereby an abstract, context- and/or position-independent phoneme /t/ is related to its allophones ([tʰ], [t], or [t˺]) that are context- and/or position-dependent. Across languages phonemes may be realised via different phones; for example, in (European) French /t/ is *not* realised as [t^h^] (Caramazza & Yeni-Komshian, 1974).

While being minimal units of lexical representation, in modern linguistic theories, phonemes are analysed as having further internal structure (i.e. comprised of phonological *features* that are defined in articulatory and/or auditory terms; Baković, 2014; Calabrese, 1988; Chomsky & Halle, 1968; Jakobson, Fant, & Halle 1951; Mielke, 2008; Stevens, 2002). That is, phonemes are bundles of features coordinated in time (to a first approximation, overlapping in time, or loosely speaking, simultaneous). A similar description is given in Fowler, Shankweiler, and Studdert-Kennedy (2016, p. 126): ‘Speakers produce phonetic segments as individual or as coupled gestures of the vocal tract,’ where there is a strong correspondence between our use of the term *feature* and their use of *gesture*. For example, the phoneme /t/ is a combination of features: [stop], which indicates that the airflow through the mouth is interrupted completely; [alveolar], which reflects a constriction at the alveolar ridge; and [voiceless], which reflects that the vocal folds are not vibrating. Allophones are often more specific realizations of phonemes which differ in the presence or absence of one or more features (e.g. [t^h^] has the additional information that it is [spread glottis]). Features can be defined in terms of both their articulatory requirements and their acoustic consequences, as illustrated for manner features in Table 1, though at times the complete definitions require multiple acoustic cues or complex quantities.

**Table 1:** Articulatory and acoustic correlates of manner features

Feature	Articulation	Acoustics
[stop]	Complete interruption of airflow	Short silent interval
[fricative]	Turbulent airflow	Aperiodic noise
[nasal]	Airflow through nose	Low-frequency resonance
[approximant]	Unimpeded airflow	Multiple resonances

The original proposal for distinctive features (Jakobson et al., 1951) emphasized the connections between articulation and audition, but other theories have seen the primary definitions of the features as articulatory (Chomsky & Halle, 1968; Halle, 1983; also articulatory phonology, Browman & Goldstein 1989) or auditory (Diehl & Kluender, 1989; Diehl, Lotto, & Holt, 2004), or as an exploitation of ‘good’ regions of articulation-acoustic convergence (e.g. quantal theory, Stevens, 1972, 1989). More recent theories, such as articulatory phonology (Browman & Goldstein, 1989; Fowler, 2015; Goldstein & Fowler, 2003), emphasize articulatory gestures as the basic ‘atoms’ of speech. But the theory also crucially involves the coordination of gestures in time (termed ‘bonding’ in Goldstein & Fowler 2003)—phonological structures of segment or larger sizes are ‘molecules’ within the theory. More importantly, for the present purposes, articulatory phonology has so far neglected to address many of the arguments that we review below; for instance, they have provided no general account of intergestural coordination coherence in resyllabification contexts (i.e. why it is that segment-sized conglomerations of gestures are resyllabified as a unit). But the theory has the relevant mechanisms to do so, as it allows for different kinds of coordination relations between gestures.^1^ Ultimately, speech is both action and perception, and we consider the original view of features as linking articulation and audition attractive and compelling (Hickok & Poeppel, 2007, 2015; Poeppel & Hackl, 2008).

In sum, although languages use different repertoires of phonemes to represent phonological forms of words, the way in which phonological forms are represented in long-term memory is thought to be universal, namely via a segment-sized, discrete, and symbolic phonemic code.^2^ Consequently, comprehending a spoken word (i.e. mapping an acoustic waveform to a phonological form which in turn provides access to the word’s meaning) necessitates mapping of the continuous acoustic signal onto a discrete phonemic code. This requires that phonemes should be retrievable from the acoustic waveform, either directly (with no recourse to features or allophones) or in a mediated way (e.g. via features and/or allophones). In this view, phonemes are access codes to the lexicon (i.e. the sublexical representations retrievable from the acoustic signal that directly interface with phonological forms of words).

In order to avoid confusion regarding our claims regarding phonemes, we should emphasize two points. First, the claim that phonemes are access codes to the lexicon does not preclude that other units may also be employed on the route of mapping an acoustic signal to a phoneme sequence. In particular, there may be independent requirements on how a speech signal is chunked that originate in considerations of echoic memory, acoustic prominence, or variability, which may demand processing unit(s) of a certain type or size. These other units coexisting with phonemes may fit into a single processing hierarchy or operate on parallel streams; the essential part that remains on the phoneme-based view is that the lexicon cannot be robustly accessed until a direct or mediated mapping from the speech signal to phonemes has taken place. Second, the critical claim behind phonemes constitutes how knowledge is stored in long-term memory rather than how this knowledge is activated during speech perception. On the phoneme-based view, there are discrete (nonoverlapping) representations devoted to each phoneme in long-term memory, but these representations can be activated in a gradient manner. For instance, the phoneme /b/ may be partially activated by the input /d/ because /b/ and /d/ share acoustic features. (A parallel from the visual word identification literature may be useful, e.g. discrete letter codes in the interactive activation model of visual word identification are activated in a continuous, graded manner; McClelland & Rumelhart, 1981.)

The hypothesis that spoken word identification involves accessing phonemes has been widely challenged in linguistics and psycholinguistics for a variety of reasons, and various alternative accounts have been advanced. In Table 2, we show a sampling of the diversity of proposals for the architecture of speech recognition from linguistics, psychology, and computer speech understanding systems. Entries within the table that do not contain ‘phoneme’ denote theories that eschew (or at least downplay severely) the role of phonemes in speech recognition.

**Table 2:** Models of speech perception, including units emphasized during signal analysis in the model, and the units used to match with stored memory representations. In many models, but not all, these units coincide (see Frauenfelder & Floccia, 1999; Pisoni & Luce, 1987, for discussion)

Units of speech perceptual analysis	Units of lexical coding	Examples
Spectra	Auditory objects	Diehl and Kluender (1987); Diehl, Lotto and Holt (2004)
Spectra	Spectra	Klatt (1979, 1980, 1989; LAFS)
Features	Features	Stevens (1986, 1992; LAFF); Lahiri and Reetz (2002)
Gestures	Gestures	Zhuang, Nam, Hasegawa-Johnson, Goldstein, and Saltzman (2009); Mitra, Nam, Espy-Wilson, Saltzman, and Goldstein (2010)
Allophones	Allophones	Lowerre (1976; Harpy); Mitterer, Scharenborg, and McQueen (2013)
Triphones (allophones with one segment of left and right context)	Triphones	Wickelgren (1969; numerous HMM models); Laface and De Mori (1990)
Allophones	Phonemes	Church (1987a, 1987b); Whalen (1991)
Robust features	Phonemes	Huttenlocher and Zue (1984)
Multiple phoneme probabilities	Phonemes	Norris and McQueen (2008)
Demi-syllable (sometimes also called ‘diphone’)	Demi-syllable	Fujimura (1976); Rosenberg, Rabiner, Wilpon, and Kahn (1983)
Syllable	Syllable	Fujimura (1975); Smith (1977; Hearsay II); Smith and Erman (1981; Noah); Ganapathiraju, Hamaker, Picone, Ordowski, and Doddington (2001); Greenberg (2006)
Word vector	Word template	Rabiner and Levinson (1981)
Fine detail	Word exemplars	Palmeri, Goldinger, and Pisoni (1993)
Fine detail & allophones	Word exemplars	Pierrehumbert (2002)

We caution that in many cases the table entries represent an oversimplification of the complete model. For example, K. W. Church (1987a, 1987b) first parses the speech input into syllables using allophones to constrain the syllabic parse, using a ‘lexicon’ of syllables for this purpose. After the syllable is recognized, its phoneme content (stored in the syllable lexicon) is then matched against the lexicon of words, which is coded in terms of phonemes. The overall matching procedure in both cases uses a lattice of possibilities, similar to a chart parser.

**Fig. 1:**
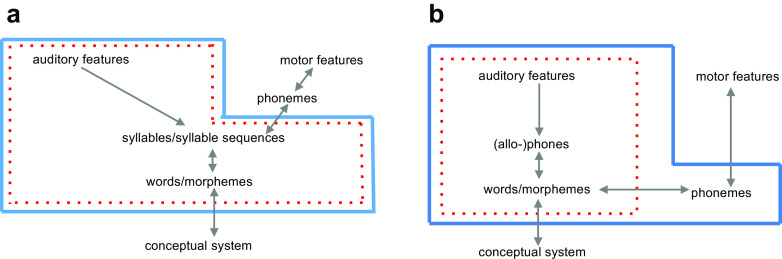
**a** Hickok’s (2014) neurocognitive model of speech processing (adopted from Hickok, 2014, with minor modifications) recruits phonemes only on the speech production route, whereas speech perception and lexical representations are assumed to operate at the level of (demi-)syllables. **b** Phonemes as postaccess codes model (Morton & Long, 1976; Warren, 1976), in which lexical representations are accessed via (allo)phones, with phoneme representations activated after a lexical representation has been retrieved. In both models, the *red dotted box* includes representations involved narrowly into speech perception/word identification, whereas a *blue solid box* includes representations available more broadly for language comprehension, including higher-level morphosyntactic and semantic computations (not shown). (Colour figure online)

In addition to the models enumerated above, some researchers have proposed models that include phonemes, but only outside of the perceptual system as part of motor preparation of possible spoken responses (e.g. Hickok, 2014; see Fig. 1a). That is, phonemes are only involved in speech production. Alternatively, phonemes are retrieved after lexical access has taken place, along with the other information such as syntactic category and semantic information (e.g. Warren, 1976; Morton & Long, 1976; see Fig. 1b). That is, phonemes are accessed postlexically but are nevertheless involved in the comprehension process.

**Fig. 2:**
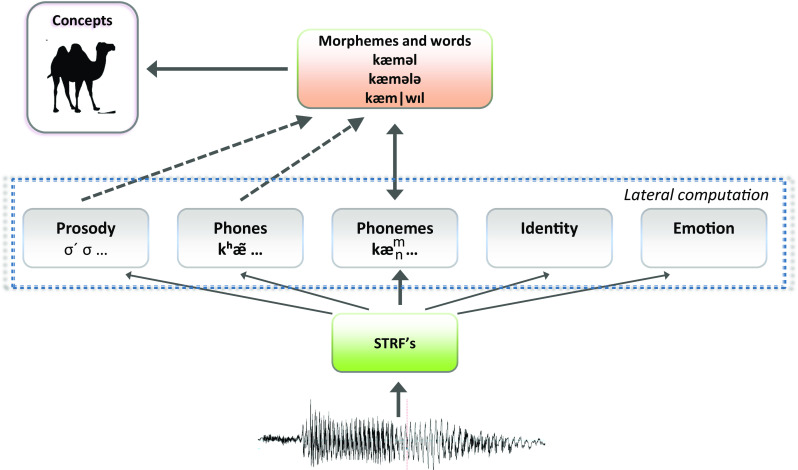
A pathway for processing a speech signal en route to word identification, exemplified for the example input *camel*. While many sources of information are extracted from the acoustic signal in parallel (see text), phonemes serve as access codes to words and morphemes

In the following sections, we argue that phonemes are essential as access codes in speech comprehension and in speech production, as highlighted by our title, ‘Phonemes: Lexical access and beyond’. We note that by placing the phoneme representations outside of the comprehension pathway, Hickok’s (2014) neurocognitive model of speech processing in Fig. 1a (see also Mehler, 1981) fails to account how listeners perform grammatical computations that require phonemes during language comprehension (which includes speech perception; see the section ‘Higher level linguistic computation’). And models where phoneme representations are retrieved postlexically for the sake of comprehension (as in Morton & Long’s, 1976, model; see Fig. 1b) fail to account for psycholinguistic and linguistic findings suggesting that phonemes play a role in speech perception. Indeed, in such a view phonemes are only accessed through a word or a morpheme, and as a consequence, there is no obvious way to create a mapping between sublexical representations (e.g. phones, syllables) and phonemes. For example, we know of no existing model such as in Fig. 1b that makes it possible to appreciate that the phones [t^h^] and [t] are allophones (i.e. representatives of the same phoneme category; we return to this issue in Part 4). In Part 2 we review psycholinguistic findings that are frequently used to reject phonemes as units of speech perception, and we show that the conclusion is unwarranted. The argument is the same in majority of cases, namely, researchers report evidence that units other than phonemes (e.g. syllables, [allo]phones, features) play a role in speech perception, and based on these findings, phonemes are rejected. However, the findings only show that phonemes are not the only sublexical phonological codes involved in perception, a claim we agree with (see Part 4 and Fig. 2). Importantly, Part 2 also discusses several psycholinguistic studies which provide positive evidence for phonemes as units of speech perception. However, the strongest evidence in our view comes from linguistic data in Part 3, which are often undeservedly ignored in the psychological literature.

## Part 2: Reconsideration of psycholinguistic challenges to phonemes

According to critics of the phoneme from speech perception (Hickok, 2014; Massaro, 1972, 1974), it is postulation of phonemes as access codes to the lexicon that leads to the lack of invariance problem (i.e. units used for lexical representation cannot be robustly recognised in the acoustic input) and/or to the linearity problem (i.e. there is no one-to-one correspondence between stretches of the acoustic signal and an ordered sequence of lexical coding units). There have been two main loci of objection to phonemes as lexical access codes: (a) size (i.e. that a phoneme corresponds to a single segment such as a consonant or vowel) and (b) abstractness (i.e. to position- and/or context-independence of the phoneme). Below we consider these two claims as well as the claim that phonemes are a by-product of literacy rather than a fundamental characteristic of spoken word identification.

### Size

One of the main challenges to the hypothesis that phonemes play an essential role in speech processing is the claim that they constitute the wrong size of unit. Rather than sublexical speech perception units being the size of a vowel or consonant, theorists argue that speech perception employs units that are larger (e.g. syllables or demi-syllables) or smaller (e.g. features) than phonemes to the exclusion of the latter.

Traditionally, the most widely accepted evidence that segment-sized elements play a role in speech processing has come from studies of naturally occurring or elicited speech errors in speech production. They demonstrate that the majority of speech errors involve insertion or deletion of a single consonant or vowel (e.g. *explain carefully* pronounced as *explain clarefully*, *same state* → *same sate*) or their exchange (e.g. *York library* → *lork yibrary*; Dell, 1986). Whereas phoneme-sized errors are ubiquitous, phonological errors rarely involve whole syllables (e.g. *napkin* → *kinnap*) or single phonological features (e.g. *blue* → *plue*; Fromkin, 1974; Shattuck-Hufnagel, 1979, 1983), which highlights a critical role of segment-sized categories in language production, because viewing whole-segment exchanges as the coincidental exchange of multiple features would vastly underpredict their relative frequency.

The role of phonemes in speech perception, on the other hand, has been challenged through arguments in favour of a larger unit such as (demi-)syllable or a smaller unit such as feature. We consider this evidence next.

#### Units of perception larger than phonemes: (Demi-)syllables

Massaro (1972, 1975; Oden & Massaro, 1978) advanced theoretical arguments in support of (demi-)syllables and against phonemes as units of speech perception (similar claims can be found in Bertoncini & Mehler, 1981, and Greenberg, 2006, among others). Massaro views spoken word identification as a bottom-up process that involves the identification of invariant (abstract) sublexical representations. From this perspective, phoneme-sized units are a poor candidate as their acoustic realisation can vary dramatically in different contexts, and so they fail the invariance criterion. For instance, the acoustics of a stop consonant is affected strongly by the following vowel: formant transitions that are part of the acoustic realisation of the consonant /d/ differ for the syllables /di/ and /du/. By contrast, the acoustics of (demi-)syllables are much less variable across contexts, leading to increased functionality of (demi-)syllables.^3^ Typically, syllables are operationalised as units of speech organisation that influence the language prosody, stress, meter, and poetic patterns and are composed of several segments (i.e. a single vowel or diphthong surrounded by zero, one, or several consonants on either side, depending on a language). Unlike this typical view, Massaro views (demi-)syllables as atomic and indivisible into segments, that is, (demi-)syllable /ku/ is stored in the long-term memory holistically without any reference to segments /k/ and /u/ (Oden & Massaro, 1978, p. 176).^4^

A key (implicit) assumption of this view is that phonemes (or, indeed, demi-syllables) are learned in a bottom-up manner. Given this premise, we agree, that the acoustic variability of phonemes may be problematic. But Massaro’s argument loses its force when phonemes are seen as linguistic units that are shaped by additional constraints in order to play a more general role in language processing. That is, if top-down constraints from words and morphemes play a role in learning sublexical representations, then the perceptual system can map together distinct acoustic versions of a phoneme to a common code. To illustrate, in the domain of visual word identification, there is widespread agreement that letters are coded in an abstract format despite the fact that there is no visual similarity (invariance) between many upper- and lowercase letters (e.g. ‘a’ and ‘A’; Bowers, Vigliocco, & Haan, 1998; Coltheart, 1981; McClelland, 1977). The lack of visual invariance is not used to rule out abstract letter codes as a unit of representation but rather is taken as evidence that top-down constraints shape letter knowledge (e.g. Bowers & Michita, 1998). The same reasoning applies to phonemes. It is perhaps worth noting that if anything the abstractions assumed for letters are more difficult, given that there is no bottom-up similarity between some upper- and lowercase letters, whereas all members of a phoneme category usually share some bottom-up similarity.

So a key question to consider when evaluating Massaro’s theoretical argument against phonemes is whether there is any independent evidence for top-down constraints on perceptual learning in speech. In fact, the evidence of top-down involvement in speech learning is robust (M. H. Davis, Johnsrude, Hervais-Adelman, Taylor, & McGettigan, 2005; Hervais-Adelman, Davis, Johnsrude, & Carlyon, 2008; McQueen, Cutler, & Norris, 2006). Indeed, even some of the most ardent supporters of modularity in the domain of online speech perception argue for top-down constraints in learning sublexical forms. For example, Norris, McQueen, and Cutler (2003) asked Dutch speakers make lexical decisions to spoken Dutch words and nonwords. The final fricative of 20 words were replaced by a sound [?] that was ambiguous between [f] and [s], and one group of listeners heard ambiguous [f]-final words (e.g. [witlo?], from *witlof*, ‘chicory’) and another group heard ambiguous [s]-final words (e.g. ambiguous [na:ldbo?], from *naaldbos*. ‘pine forest’). Listeners who had heard [?] in *f*-final words were subsequently more likely to categorize ambiguous syllables on an /ef/ – /es/ continuum as [f] than those who heard [?] in *s*-final words, and vice versa. That is, participants altered the boundary of the phonemes to be consistent with its lexical context (e.g. participants learned that ambiguous [?] presented in [f]-final words was a strange way to pronounce [f]). The important implication for present purposes is that the rejection of phonemes based on the lack of acoustic invariance is misguided because the invariance need not be present in the bottom-up signal. To be clear, the evidence for top-down learning does not by itself provide evidence for phonemes (top-down influences could contribute to all forms of sublexical representations), but it does undermine a common argument against phonemes (e.g. Massaro, 1972).

In addition, three empirical findings are often used to support the conclusion that syllables rather than phonemes constitute the sublexical representational units involved in spoken word identification. First, Massaro (1975; Oden & Massaro, 1978) note that some consonants cannot be perceived in isolation from their syllable context. For example, a gradual removal of the vowel from the consonant-vowel (CV) syllable /da/ does not result into a stimulus which is heard just as /d/. Rather, the listener continues to perceive the CV syllable until the vowel is eliminated almost entirely, at which point a nonspeech chirp is heard (Mattingly, Liberman, Syrdal, & Halwes, 1971). This would be a strong argument for syllables rather than phonemes on the premise that all perceptual units should support *conscious* perception. But if phonemes are abstract codes that interface with lexical knowledge in the service of word identification and other linguistic computation, then it is misguided to rule out phonemes based on a limited introspective access to them. To provide a parallel from written representations, the fact that readers can perceive an uppercase ‘A’ or lowercase ‘a’ but do not have an awareness of an abstract A* does not suggest that there are no abstract letter codes. Similarly, the fact that listeners cannot hear phonemes in isolation should not be used to rule out phonemes.

Second, Massaro (1974) used masking experiments to determine that the temporal span of preperceptual auditory storage is about 250 ms. He argued that perceptual units in speech should be organized around this temporal window, opting for (demi-)syllables. Note, however, that the size of the preperceptual auditory storage suggests that sublexical phonological codes are not larger than a syllable, but it provides no evidence against phonemes. In particular, the preperceptual storage may hold a sequence of multiple perceptual units (i.e. multiple phonemes).

The third piece of evidence comes from perceptual monitoring experiments such as Savin and Bever (1970), in which participants listened to a sequence of syllables (e.g. *thowj*, *tuwp*, *barg*) and had to identify as quickly as possible whether it contained a certain phoneme (e.g. /b/) or syllable (e.g. *barg*). Response times were consistently faster for syllables compared to phonemes (subsequently replicated by Foss & Swinney, 1973; Segui, Frauenfelder, & Mehler, 1981; Swinney & Prather, 1980), leading to the inference that phonemes are identified after syllables. On this basis Savin and Bever (1970) reject phonemes as access codes to words (although they highlight indispensability of phonemes for other linguistic computations).

However, Savin and Bever’s (1970) simple conclusion has been challenged. From a methodological point of view, the syllable-over-phoneme advantage was argued to be an artefact of experimental stimuli used in earlier studies (McNeill & Lindig, 1973; Norris & Cutler, 1988); for example, Norris and Cutler (1988) showed that it disappears when a detailed analysis of the stimulus is required in order to perform correctly on both ‘yes’ and ‘no’ trials. More importantly, a conceptual problem has been pointed out: The advantage of syllables over phonemes might not reflect the fact that syllables are accessed first in speech perception, but rather that participants have a faster introspective access to them (e.g. Foss & Swinney, 1973; Healy & Cutting, 1976; Rubin, Turvey, & Van Gelder, 1976; Segui et al., 1981). The idea that conscious introspection is dissociated from online processing has a long history in other domains (e.g. vision). For example, according to Ahissar and Hochstein’s (2004) reverse hierarchy theory, visual perception involves activating a series of representations organised in a hierarchy from bottom up. Yet conscious perception begins at the top of the hierarchy (where information is coded in an abstract fashion) and moves to lower levels (where information is coded in a more specific manner) as needed. Applying the same logic to speech (Shamma, 2008), earlier conscious access to syllables over phonemes is not the basis for concluding that phonemes are strictly processed after syllables, or that syllables are access codes to the lexicon to the exclusion of phonemes.

Moreover, listeners are able to perform phoneme monitoring in nonwords (Foss & Blank, 1980), sometimes even showing a nonword advantage (Foss & Gernsbacher, 1983). This shows that a phoneme representation can be constructed without an existing lexical item, so then one possibility is that the phoneme content of syllables is retrieved when identifying a syllable (as in K. W. Church, 1987a, 1987b). However, listeners are also able to perform phoneme monitoring when the target is embedded within an illicit syllable in the language (Weber, 2002). Thus, they do not just rely on an auxiliary lexicon of the attested syllables of their language. More generally, as noted by an anonymous reviewer, phoneme monitoring in languages with an alphabetic script may not be a purely phonological task and may involve accessing orthographic information as well.

To summarize thus far, the above theoretical and empirical arguments taken to support syllables as opposed to phoneme representations are weak, and indeed, the findings can be readily accommodated by a theory that incorporates both phonemes as well as syllables. More importantly, there are also empirical findings that lend direct support for the conclusion that segment size units play a role in speech perception, as detailed next.

One strong piece of evidence in support of phonemes comes from artificial language learning studies that exploit listeners’ ability to learn language on the basis of statistical regularities. In a typical experiment, listeners are first exposed to a continuous speech stream devoid of any intonational cues or pauses which (unbeknown to the listeners) is constructed of several nonsense words—for example, the stream.... *pabikutibudogolatudaropitibudopabiku*…based on words *pabiku*, *tibudo*, *golatu*, and *daropi* (Saffran, Aslin, & Newport, 1996; Saffran, Newport, & Aslin, 1996; Saffran, Newport, Aslin, Tunick, & Barrueco, 1997). Whereas initially listeners perceive the stream as a random sequence of individual syllables, they become able to segment out words after several minutes of exposure, on the basis of transitional probability (TP) from one syllable to the next, which is higher for syllables within words than for syllables across word boundaries (1 vs. 1/3 in the example above). This finding demonstrates that syllables are accessible to the perceptual system as units over which statistical computations can be made. The question is then whether similar computations can be performed over phonemes.

The critical evidence that similar statistical inferences can be made at the phoneme level comes from studies by Newport and Aslin (2004); Bonatti, Peña, Nespor, and Mehler (2005); Toro, Nespor, Mehler, and Bonatti (2008), and others. In these studies participants listened to a continuous stream containing nonsense words from several ‘root families’, each based on a triconsonantal root-mimicking aspects of Semitic languages—for example, roots *p*_*r*_*g*_, *b*_*d*_*k*_ or *m*_*l*_*t*_ that were combined with different vowels to produce four words in each family (e.g. *puragi*, *puregy*, *poragy*, and *poregi* for the *p*_*r*_*g*_ family; Bonatti et al., 2005). Following an exposure to a continuous stream such as…*puragibydokamalituporagibiduka*…, participants could learn the consonantal roots used in the stream (as measured by their ability to choose a word such as *puragi* over a partword such as *ragiby* in the test phase). This outcome could not be achieved via tracking TPs between syllables, which were the same for syllables within and across word boundaries and instead required tracking TPs between consonants that were higher within-word than across word boundaries. The parser’s ability to track statistical regularities between nonadjacent consonants (or vowels) clearly demonstrates that segment-sized units are functional in speech perception.^5^

A similar conclusion can be reached on the basis of the findings by Chambers, Onishi, and Fisher (2010), who trained participants using nonword CVC syllables in which each consonant only appeared before or after certain vowels. For example, participants were trained on /b/ -initial syllables (e.g. /bεp/, /bis/). In the subsequent test, participants were quicker to repeat novel syllables that followed the pattern whether they had the same vowel as the one used in training (e.g. /bεs/) or a novel vowel (e.g. /bus/) as compared to syllables that violated the pattern (e.g. /b/ in the final position, as in /pεb/ or /sub/, respectively). Therefore participants could learn that particular consonants occurred as onsets (e.g. ‘b is in the onset of the syllable’), a generalisation that requires ability to operate consonants independent of vowels and is unavailable if perception operates on (holistic) syllables but not segments.

Another important piece of evidence in support of segments in speech perception is provided by phonological fusions—that is, incorrect responses given by listeners reporting the stimulus from the target ear in a dichotic listening task (Cutting, 1975; Cutting & Day, 1975). For example, the presentation of *banket* into the target ear and *lanket* into the other ear yields misreports such as *blanket*; similarly, *pay*–*lay* pair yields misreports such as *play*, *go*–*row* yields *grow*, and *tass*–*tack* yields *tacks* or *task*. As argued by Morais, Castro, Scliar-Cabral, Kolinsky, and Content (1987), these phonological fusions provide strong evidence for segment-sized units in speech perception: If syllables were the smallest perceptual unit, it would remain unclear how and why two CVC inputs (*ban* and *lan*) would result in the perception of a CCVC syllable *blan* (rather than combine into a CVCCVC string *banlan* or *lanban*).

To summarize, we have challenged theoretical and empirical arguments used to reject segment-sized perceptual units in favour of larger sublexical units and provided empirical evidence for segment-sized units in speech production and perception.

#### Units of perception smaller than phonemes: Features

In another line of research phonemes are rejected in favour of smaller units of speech perception, namely, features. Typically this research finds empirical evidence for features and concludes that phonemes are superfluous. By contrast, we argue that while features are real, they exist as internal constituents of phonemes but cannot replace phonemes.

Consider again Hickok’s (2014) model, which incorporates features and syllables but not phonemes as units on the speech perception route (see Fig. 1a). In this view, auditory features are recognised in the speech signal and then groups of features are mapped onto a syllable, with syllables being access codes to words. Each syllable is thus represented as a conglomeration of acoustic features–for example, /pu/ corresponds to {stop, labial, voiceless, vowel, high, back}. (Although we use conventional feature names that are of articulatory origin which familiar to the general readership, in Hickok, 2014, the features extracted from the acoustic input are of acoustic nature, i.e. the list above corresponds to {transient, diffuse grave, voiceless, resonant, low F1, low F2}.) Note that because the syllable /pu/ is indivisible (i.e. it does not correspond to a combination of phonemes /p/ and /u/), the feature list that corresponds to the syllable is essentially unordered (i.e. there is no mechanism posited to group the first three features—or equally, the last three features—as belonging together as a coherent unit; the features are not coordinated in time below the syllable). However, an unordered set of features makes it impossible to distinguish consonant orders within syllable codas, incorrectly resulting in identical feature lists for pairs such as /mask/ in *mask* versus /maks/ in *Max*. Introducing more structure to a syllable’s feature list admits the necessity to bundle features (i.e. it eventually recreates phonemes). As another example, consider the coda [pst] as in *lapsed*, which on the phoneme-based view is represented as the sequence of three phonemes—that is, /p/ represented as {stop, labial, voiceless}, /s/ represented as {fricative, alveolar, voiceless}, and /t/ represented as {stop, alveolar, voiceless}. In order to yield the output [pst], the timing of the switch from stop to fricative must coincide with the switch in place from labial to alveolar; otherwise, a spurious output such as [pft] may be obtained, /f/ being {fricative, labial, voiceless}. Hence again, a coordinated bundling of features into phonemes cannot be dispensed with.

A similar point can be made on the basis of the phonological fusion data by Cutting (1975), discussed in the section above. The crucial observation is that the blending process necessarily retains phonemes from the input (i.e. the acoustic features coordinated in time and comprising segments are retained as such). The acoustic features are not combined into a single, different segmental percept, though such combinations are featurally possible, that is, *pay*–*lay* pair yields *play* but not *way*, even though the labial approximant /w/ combines acoustic features of /p/ and /l/.^6^

Mesgarani, Cheung, Johnson, and Chang (2014; see also Shamma, 2014) report neurophysiological evidence for features which they tentatively use to relegate phonemes to the sidelines: ‘A featural representation has greater universality across languages, minimizes the need for precise unit boundaries, and can account for coarticulation and temporal overlap over phoneme based models for speech perception’ (p. 1009). However, such a conclusion downplays the significance of some of their own findings that lend support to phonemes. In particular, they found varying degrees of specificity in the cortical responses in the human auditory cortex, from sites that respond to a single feature to sites that conjunctively code for feature combinations such as [stop] & [labial] or [stop] & [voice]. Inspection of their Fig. 2a shows at least one site which is selective to the phoneme /b/. The existence of neurons selective for individual features and others that are selective to conjunctive feature coordinations suggests that features are coordinated during speech perception, that is, for phonemes (although it is worth noting the limited amount of evidence of this sort to date).

To summarize, there is well-accepted evidence for segments in speech production, growing evidence for segment-sized units in perception, and fundamental flaws in the arguments that are commonly put forward against segment-sized units. We conclude that segment-sized units play a role in both speech production and perception.^7^ We next consider whether these units are abstract in a manner consistent with the definition of phonemes.

### Abstraction

In addition to challenging phonemes on the basis of their size, researchers have questioned the claim that speech perception involves abstract representations. On traditional phonological theories, words are represented in long-term memory as sequences of phonemes (Lahiri & Marslen-Wilson, 1991; Lahiri & Reetz, 2002; Stevens, 2002) and spoken word identification involves a perceptual normalization process aimed at identifying phonemes while filtering out acoustic variability that is not strictly relevant for identifying words. One source of acoustic variability is due to the presence of indexical information that characterizes both the speaker (the speaker’s sex, accent, age, identity, emotional state, etc.) and the physical or social context in which words are spoken (e.g. type of background noise or social interaction). Another source of acoustic variability that we will refer to as ‘fine phonetic detail’ is language-internal and includes variation in the realisation of a segment depending on the nature of neighbouring segments, its position within a syllable or word, and so on.

In contrast with the normalization processes involved in identifying phonemes in traditional theory, episodic theories of speech perception claim that much or all the above variability remains in the sublexical and lexical representation, and this variability plays a functional role in word perception (Johnson, 1997; Port, 2007, 2010a, 2010b). In this view, word identification involves matching an incoming acoustic waveform to a detailed stored speech representation rather than abstract phonemes (or for that matter, abstract syllable representations). As put by Port (2007),

words are not stored in memory in a way that resembles the abstract, phonological code used by alphabetical orthographies or by linguistic analysis. Words are stored in a very concrete, detailed auditory code that includes nonlinguistic information including speaker’s voice properties and other details. (p. 143)

Empirical evidence for the claim that spoken word identification involves accessing acoustically detailed rather than abstract phoneme representations comes from demonstrations that indexical information and fine phonetic details impact on word identification. In what follows we argue that indexical and fine phonetic detail, respectively, can indeed impact on word identification, but nevertheless, there is no reason to reject the hypothesis that phonemes are abstract.

#### Indexical information

A commonly used method to assess the impact of indexical or environmental variation on spoken word identification is long-term priming. In this procedure, participants listen to a series of (often degraded) words during a study phase and later (typically with delays ranging from a few minutes to hours) the words are repeated along with a set of new control words. Priming is obtained when repeated words are identified more quickly or more accurately than nonrepeated control items (even without explicit memory for the study items; Graf & Schacter, 1985).

The critical finding for present purposes is that the size of the priming effects for repeated words is often reduced when the words differ in their indexical details between study and test. For example, Schacter and Church (1992) reported that a change of speaker resulted in reduced priming in an identification task for test words degraded with a white noise mask (see Goldinger, 1996; Sheffert, 1998, for similar results). Similarly, B. A. Church and Schacter (1994) found that changes in the speaker’s emotional or phrasal intonation or fundamental frequency all reduced priming for test words degraded with a low-pass filter. More recently, Pufahl and Samuel (2014) found reduced priming when degraded words were repeated with different environmental sounds at study and test (e.g. a phone ringing at study, dog barking at test).

There are, however, both theoretical and empirical reasons to be cautious about rejecting phonemes based on these types of findings. With regards to the empirical findings, the impact of indexical variation on priming is quite mixed. For example, in contrast to the voice specific priming effects observed in younger adults, voice-independent priming effects have been observed in elderly participants (Schacter, Church, & Osowiecki, 1994) or in patients with amnesia (Schacter, Church, & Bolton, 1995). That is, voice specific effects were lost in individuals with poor episodic memory, leading the authors to suggest that voice-specific and voice-invariant priming may be mediated by different memory systems. That is, voice-specific priming observed in young participants reflects contributions from their intact episodic memory system, whereas voice-invariant priming in the elderly and amnesic subjects reflects memory in the perceptual system that provides core support for word identification. Consistent with this hypothesis, Luce and Lyons (1998) found that the effects of indexical information on priming are lost in younger participants when repeated test words are presented in the clear in a lexical decision task (rather than degraded in some fashion in an identification task), and Hanique, Aalders, and Ernestus (2013) showed that specificity effects reemerge in the lexical decision tasks when a higher percentage of items are repeated at study and test. That is, specificity effects in priming tasks are largest under conditions in which episodic memory may play a larger role in task performance. It is also important to note that in most spoken word priming studies, the delay between study and test does not include a night of sleep that is often claimed to be important for consolidating new memories into the lexical system (Dumay & Gaskell, 2007). This also suggests that the observed, indexical effects on priming may reflect episodic memory processes that are separate from the speech perception system.

Attributing indexical effects to episodic memory is not the only way to reconcile these effects with abstract phonemes. Another possibility is that the acoustic signal is processed in two parallel streams, with a left-lateralized stream dedicated to extracting abstract phonemes, and another one (perhaps right-lateralized) that processes more detailed acoustic representations so that the listener can use indexical information in adaptive ways, such as identifying the speaker based on their voice or the emotionality of speech (Wolmetz, Poeppel, & Rapp, 2010). Indeed, there is a variety of neuropsychological evidence consistent with the hypothesis that the acoustic input is analysed in abstract and specific channels separately, and that the two systems can be doubly dissociated following left and right hemisphere lesions (Basso, Casati, & Vignolo, 1977; Blumstein, Baker, & Goodglass, 1977). In either case, indexical effects are not inconsistent with phonemes (for similar conclusions, see Cutler, 2008).

#### Fine phonetic detail

Similarly, it is premature to reject phonemes on the basis of studies showing that word identification is influenced by fine phonetic detail, as the term *fine phonetic detail* encompasses several types of acoustic variability that emerges due to language-internal factors. Below we break down findings of how fine phonetic detail affects word identification into three types: (a) prototypicality effects, (b) effects of fine phonetic detail stemming from phoneme variation due to neighbouring segments, or (c) position within a word or syllable.

#### Prototypicality effects across acoustic realisations

Even when the speaker, word, or context are fixed, segments have a range of admissible acoustic realisations, with some tokens being more frequent or prototypical than others (e.g. Lisker & Abramson, 1964; Peterson & Barney, 1952). For example, the English voiceless labial stop /p/ features the voice onset time (VOT) anywhere in the range between 15 and 100 ms, with 30 ms VOT being the most typical value; the VOT range for its voiced counterpart /b/ is −130 to 0 ms, with 0 ms being most typical. Prototypicality effects in speech perception have sometimes been taken as a challenge to phonemes. For instance, in McMurray, Tanenhaus, and Aslin’s (2009) ‘visual world’ eye-tracking study, participants heard a target word (e.g. *bear*) while looking at a visual display containing an image of a bear and an competitor image of a pear. The VOT of the initial consonant of the target varied such that although the segment always fell within the /b/ category, some VOT values were prototypical of /b/ and others closer to the b/p categorical boundary. Participants gave more looks to the picture of a pear as the VOT of the initial consonant approached the categorical boundary, which was taken as evidence that fine-grained phonetic differences within a phonemic category impact on word identification. (For similar conclusions based on other typicality effects, including vowel typicality, see Bürki & Frauenfelder, 2012; McMurray, Aslin, Tanenhaus, Spivey, & Subik, 2008; Trude & Brown-Schmidt, 2012. See also Andruski, Blumstein, & Burton, 1994, for prototypicality effects in semantic priming).

Yet it is unclear how these findings challenge phonemes. Finding of graded effects of prototypicality can easily be explained via a reasonable premise that the normalization procedure for phonemes takes more effort as the acoustic input becomes less prototypical. Alternatively, as pointed out in Part 1, nonprototypical exemplars may partially activate nontarget phonemes, leading to graded effects. At any rate, positing abstract phonemes in no way leads to the prediction that all of its acoustic realisations provide equally easy access to the phoneme, and accordingly, many findings of subphonemic details impacting on word identification have little bearing on the question of whether phonemes exist.

#### Contextual variants of phonemes: Effects of phoneme variability due to neighbouring segments

Neighbouring segments may affect acoustic realisation of a phoneme in a graded or categorical way.^8^ Graded effects are often due to coarticulation (e.g. in American English, vowels preceding a nasal consonant may be nasalised to a varying degree, as in *ham*, *ban*; Cohn, 1993). Categorical effects of segmental environment include allophonic variation (which may or may not originate in mechanical constraints on articulators), for example, English consonants /g/ and /k/ are realised as a palatalized [g^j^] before front vowels as in *geese*, *gill* or a velarized [g^γ^] before back vowels as in *goose*, *gum* (Guion, 1998). On traditional phonological theories such, contextual variability is normalized for on the route to assessing phonemes. By contrast, on many instance-based theories, acoustic variability is a key component of the sublexical representation that supports word identification, and, accordingly, no normalization process is required.

A key theoretical motivation for using finer-grained variants of phonemes as perceptual units is their greater acoustic stability compared to phonemes themselves, which is thought especially critical for the acquisition of phonology (Pierrehumbert, 2002, 2003). Yet the argument for positional variants of phonemes as perceptual units rests on the same implicit (and unwarranted) assumption that Massaro adopted when arguing for (demi-)syllables (see the section ‘Units of perception larger than phonemes: (Demi-)syllables’, above), namely that sublexical perceptual units must code for portions of speech that are *acoustically* invariant. However, as we argued earlier, involvement of top-down knowledge in shaping sublexical categories enables mapping dissimilar acoustic patterns to common sublexical representations.

Empirical evidence for the existence of context-specific variants of phonemes is abundant, and often taken as a challenge to phonemes. For example, Reinisch, Wozny, Mitterer, and Holt (2014) conducted a perceptual learning study which trained participants to identify a novel degraded or distorted speech sound as an allophone of some phoneme in one context and assessed whether learning generalizes to a different context. It is assumed that generalization should scope over all other allophones of that phoneme if phonemes indeed play a role in speech perception. However, the authors found that learning to categorize an ambiguous [b/d] sound in the context of the vowel /a/ as either /b/ or /d/ did not generalize to /u/ context, despite similarities of acoustic encoding of the /b/ vs. /d/ distinction in both contexts, leading to the conclusion that prelexical processing does not make use of context-free phonemes. Dahan and Mead (2010) report similar findings, although, notably, they are more cautious in using them to argue against the phoneme view.

Other studies demonstrate effects of subphonemic durational and/or prosodic variation on speech segmentation and word identification (Cho, McQueen, & Cox, 2007; M. H. Davis, Marslen-Wilson, & Gaskell, 2002; Gow & Gordon, 1995; Salverda, Dahan, & McQueen, 2003; Salverda et al., 2007). In Salverda et al.’s (2003) eye-tracking visual-world paradigm study, listeners heard an auditory target word (e.g. /hamster/), cross-spliced so that the first syllable /ham/ was replaced either by a recording of the monosyllabic word *ham* or by the first syllable from a different recording of the word *hamster*. Listeners had more transitory fixations to the monosyllabic competitor picture *ham* in the former than latter condition, which was taken as evidence against abstract phonemes being used for word representation and identification (e.g. Salverda et al., 2007). Similarly, coarticulatory effects on word identification were also taken as incompatible with phonemes. Dahan, Magnuson, Tanenhaus and Hogan (2001) found that listeners identified the object ‘net’ more slowly from a cross-spliced acoustic input *ne*_*k*_*t* that combines the syllable onset *ne*_*k*_ extracted from *neck* with the coda *t* extracted from *net* than when the acoustic input *ne*_*t*_*t* was still cross-spliced but contained no coarticulatory mismatches (see also Marslen-Wilson & Warren, 1994; McQueen, Norris, & Cutler, 1999). We note, however, that the fact that the consonant /t/ is normally realised both in the formant transitions of the preceding vowel and in the consonant closure/release. In *ne*_*k*_*t* only the closure but not the formant transitions carry the information on /t/, thus delaying the identification of ‘net’.

The findings above clearly demonstrate that subphonemic details can have an effect on perceptual learning and spoken word identification. But contrary to the authors’ conclusion the results do not provide any evidence against phonemes, in particular, against models in which both context-specific phones and phonemes play a role in speech perception. To illustrate our point, consider the finding that even more acoustically specific effects can be observed in speech perception (e.g. perceptual learning is sometimes ear specific; Keetels, Pecoraro, & Vroomen, 2015). Clearly, it would be inappropriate to reject allophones on the basis of ear-specific learning, and in the same way, it is inappropriate to reject phonemes on the basis of allophone-specific learning. The simple fact is that all forms of representations can coexist, and accordingly, evidence for one sort of representation does not constitute evidence against another.

To summarize, once again, the above theoretical and empirical arguments taken to challenge phoneme representations are weak, and, indeed, the findings can be readily accommodated by a theory that incorporates both phonemes as well as other sublexical units of representation. Hence, while we agree with the claim that context-specific variants of phonemes play a role in acquisition (as in Pierrehumbert 2002, 2003) and speech segmentation/word identification, this conclusion provides no evidence against with phonemes. Furthermore, there are empirical findings that we discuss next, that lend direct support for the conclusion that abstract segment-sized units play a role in speech perception.

#### Positional variants of phonemes: Variability across syllable or word position

Another key characteristic of phonemes is that they are independent of syllable or word position (i.e. the same /b/ phoneme is used as an access code for *book* and *tab*). Indeed, position-independent phonemes are widely accepted for speech production (Bohland, Bullock, & Guenther, 2010; Guenther, 2016). Often-cited evidence for phonemes in language production comes from speech errors in segments exchange. Although the bulk (89.5%) of exchanges are bound by syllable position (e.g. syllable onset exchanges as in *York library* → *lork yibrary*, *left hemisphere* → *heft lemisphere*; Dell, 1986), there is a small but nonneglectable amount of exchanges across syllable positions (e.g. *film* → *flim*; Vousden, Brown & Harley, 2000). More recent support comes from Damian and Dumay’s (2009) picture-naming study in which English speakers named coloured line drawings of simple objects using adjective-noun phrases. Naming latencies were shorter when the colour and object shared the initial phoneme (e.g. *green goat*, *red rug*) than when they did not (*red goat*, *green rug*). Critically, facilitation was found even when the shared phoneme switched its syllable/word position (e.g. *green flag*). As acoustic realisation of the same phoneme (/g/ in the last example) varies by position, the facilitatory effect cannot be fully attributed to motor-articulatory planning and supports abstract position-independent representations in speech production. For further empirical evidence, see Reilly and Blumstein (2014).

On the speech perception side, however, the claim that position-independent sublexical units play a role in spoken word identification is often rejected. One issue is theoretical; namely, it is not obvious how to code for order of phonemes if the representations themselves are position independent. For example, in order to identify the word *cat*, it is not sufficient to identify the phonemes /k/, /æ/, and /t/, given that these three phonemes can also code for the word *act*. Indeed, as far as we are aware, there are no existing algorithmic models of spoken word identification that explain how position-independent phoneme representations are ordered in order to distinguish words with the same phonemes in different orders.

Instead of positing position-invariant phonemes, theorists tend to assume that segments are coded differently when they occur in different contexts and positions within words. For example, Wickelgren (1969, 1976) represents words via context-sensitive allophones that encode a segment in the context of the preceding and the following segments. So the word *cat* is represented via the set of allophones /_#_k_æ_/, /_k_æ_t_/, and /_æ_t_#_/, and *act* is represented by the allophones /_#_æ_k_/, /_æ_k_t_/, and /_k_t_#_/, which leads to no ambiguity between the sets representing *cat* and *act*. More commonly, it is assumed that segments include subphonemic information that help specify the order of the segments (e.g. the segment /b/ has X feature when it occurs in the onset, and Y feature when it occurs in the coda position of a syllable). What we would emphasize here is that in both cases theorists are rejecting position-invariant phonemes and are replacing them with more detailed representations that code for both identity of a segment and its order.

It is important to note, however, that the there are ways to code for order using position-independent phoneme representations. Indeed, in the visual word-recognition literature, a similar issue arises regarding how to order letters, and both context-specific (e.g. representing letters by position or by surrounding letters; Grainger & Van Heuven, 2003) and position-independent (C. J. Davis, 2010) letter codes have been proposed and implemented in algorithmic theories. Leaving aside an evaluation of (dis)advantages of the different coding schemes, the main point is that solutions for encoding order on the basis of position-independent letter codes exist, and the solutions might be adapted to the problem of ordering position invariant phonemes. Accordingly, theory does not rule out position invariant phonemes, and the key question is whether position-specific or invariant units provide a better account of the empirical data in psychology and linguistics.

Turning to empirical literature, support for the hypothesis that speech perception is mediated by position-specific allophones comes from perceptual learning studies (Dahan & Mead, 2010; Mitterer, Scharenborg, & McQueen, 2013; Reinisch, Wozny, Mitterer, & Holt, 2014; see the section above for task description). Mitterer et al. (2013) successfully trained listeners to classify a novel morphed sound as the acoustic realisation of either the phoneme /r/ or /l/ in the final position, but this learning did not affect perception of syllable-initial allophones of /r/ or /l/, leading to the conclusion that perceptual learning—and by extension speech perception—is mediated by position-specific allophones rather than phonemes. Yet it is unclear why altering the perceptual space of the final allophones of /r/ or /l/ via training should also affect the perceptual space associated with initial allophones (that may be acoustically rather distinct from the final allophones). To briefly illustrate, assume that there are indeed abstract visual letter codes that map together ‘A’ and ‘a’ to a common code. If perceptual learning led a reader to expand the perceptual category of capital ‘A’ (e.g. expanding it to a decorative variant ‘’), there is no reason to expect that the perception of ‘a’ has been in any way altered. In the same way, the absence of generalisation from one allophone to another is expected on any account, and accordingly, this observation does not serve as evidence against phonemes in speech perception (for more detail, see Bowers, Kazanina, & Andermane, 2016).

Another source of support for position-specific (allo-)phones is provided by selective adaptation studies (Ades, 1974; Samuel, 1989). For example, Ades (1974) found that listeners’ categorical boundary in the /dæ/ – /bæ/ continuum shifted towards /bæ/ following adaptation with a syllable-initial /d/ (as in /dæ/), but not following adaptation with a syllable-final /d/ (as in /æd/). The finding that the syllable-final, unreleased allophone [d̚] in the adaptor /æd/ had no effect on the perception of a syllable-initial, necessarily released allophone [d] was taken to suggest that the speech-perception system treats the initial and final *d*s separately, as opposed to position-invariant phonemes.

We would note two points undermine the common rejection of position-invariant phonemes based on the above studies. First, as highlighted above, theories that posit phonemes do not reject other sublexical representations, and, indeed, allophones are central to phonological theories. Accordingly, obtaining evidence *for* allophones is in no way evidence *against* phonemes, merely that the task was viewed as being more relevant to phones. Second, a number of studies provide positive evidence in support of position-invariant phonemes. For example, a recent selective adaptation study by Bowers et al. (2016) obtained just the opposite findings from Ades (1974) and Samuel (1989). Bowers et al. used adaptor words that either shared a phoneme /b/ or /d/ in the initial position (e.g. *bail*, *blue*, *bowl*) or a final position (*club*, *grab*, *probe*). The listeners then judged an ambiguous target *b*/*dump* (produced by morphing the words *bump* and *dump*). A significant adaptation effect was found both with initial and final adaptors (i.e. the target *b*/*dump* was identified as ‘dump’ more often following /b/ -adaptors than /d/ -adaptors in both conditions, leading to the conclusion that position-independent phonemes are involved in speech perception). Further evidence for position-independent phonemes in speech perception comes from Toscano, Anderson, and McMurray’s (2013) study using the visual-world paradigm on anadromes (i.e. reversal word pairs such as *desserts* and *stressed*, or *bus* and *sub*). Listeners showed more fixations to anadromes (e.g. *sub* when *bus* is the target) than either to unrelated words (*well*) or to words that share fewer phonemes (*sun*). This finding cannot be accounted for via perceptual units such as (demi-)syllables (as *sub* is no closer to *bus* than *sun* is) or via phones (as at this level *sub* is farther from *bus* than *sun*) but can be naturally explained in terms of phonemes (as *sub* and *bus* share all of the phonemes). Finally, Kazanina, Phillips, and Idsardi (2006) demonstrate that early perceptual MEG responses to the same pair of nonsense syllables, [da] and [ta], is modulated by whether their initial consonants are separate phonemes (as in English or Russian) or allophones of the same phoneme (as in Korean). The finding that early stages of speech perception (within 150–200 from the sound onset) are affected by the phonemic status of the sounds strongly suggests that phonemes are units of speech perception.

To summarise the section ‘Abstraction’, indexical or fine phonetic details can impact word identification under some conditions, and it is uncontroversial that listeners can perceive and use such information for the sake of communication more broadly construed (e.g. Local, 2003). Yet the question is whether these findings falsify the claim that abstract phonemes are a key component of spoken word identification and speech processing more generally. In our view, the answer is a clear ‘no’. The representations responsible for the above indexical or fine phonetic detail results may coexist with abstract phoneme representations (cf. Cutler, Eisner, McQueen, & Norris, 2010; Pisoni & Tash, 1974).

### Phonemes are outcomes of literacy

Even if the above criticisms of phonemes are rejected, and the (allegedly limited) psycholinguistic evidence in support of phonemes accepted, it is possible to raise another objection, namely, phonemes are an artificial by-product of literacy and accordingly do not constitute a core component of speech recognition. (Similarly, Greenberg, 2006, identifies alphabet-based orthography as the culprit for why phonemes are considered as units of speech perception in the first places.) And indeed, most studies that are taken to support phonemes are carried out in literate adults, as are the vast majority of adult psychological studies. Furthermore, there are demonstrations that preliterate children have difficulty identifying the number of phonemes but not syllables in a word (I. Y. Liberman, Shankweiler, Fisher, & Carter, 1974), and demonstrations that illiterate adults have difficulties in tasks that require explicit manipulation of phonemes, such as deleting the initial consonant from a spoken word (Lukatela, Carello, Shankweiler, & Liberman, 1995; Morais, Bertelson, Cary & Alegria, 1986; Morais, Cary, Alegria & Bertelson, 1979). In nonalphabetic languages such as Mandarin Chinese, even literate speakers often show a lack of phoneme awareness on explicit tasks (Read, Zhang, Nie, & Ding, 1986). Together, these findings at least raise the possibility that phonemes only exist as a by-product of learning an alphabetic script.

Another possible interpretation of these findings, however, is that exposure to an alphabetic writing system highlights the role of preexisting phoneme representations, making phonemes more consciously accessible and more easily manipulated for literate individuals. Indeed, when the requirement for explicit report is removed, illiterate listeners performance shows evidence for phonemes. For example, Morais, Castro, Scliar-Cabral, Kolinsky, and Content (1987) tested literate and illiterate Portuguese speakers in a dichotic listening task similar to the one in Cutting and Day (1975; see the section ‘Units of perception larger than phonemes: (Demi-)syllable’, above) and reported phonological fusions that involved a single segment for both groups (although the proportion was higher in the literate than illiterate group, 52% vs. 37%). Phonological fusions involving migration of a single consonant were also found (e.g. the input pair /pal∧/ – /bɔdə/ yielded /baldə/). Such phonological fusions and other evidence—including the fact of emergence of alphabetical systems in the human history in the first place (see Fowler, 2015) support the claim that abstract segment-sized units of perception are not uniquely a by-product of learning a written alphabet, although they become more accessible for metalinguistic awareness via orthography.

Last but not least, we point out that many linguistic computations that require phoneme units are present in illiterate adults and in children (e.g. see the section ‘Alliteration in poetry’, below).

To conclude Part 2, current psycholinguistic data are consistent with the hypothesis that syllables, features, indexical, fine phonetic detail, as well as phonemes may all have a role in spoken word identification. There is no reason to reject phonemes on the basis that additional representations may be involved in word identification.

One possible criticism to our claim that evidence for segments, phones, and syllables does not rule out phonemes is that we have rendered phonemes unfalsifiable. We have two responses to this. First, there has never been a theory in which phonemes constitute the only sublexical representation, so it is just a logically invalid conclusion to reject phonemes based on evidence *for* syllables. That is, there is at least a further assumption of an Ockham’s razor for the argument to go through. The fact that there is some positive evidence in support of phonemes from the psycholinguistic literature (e.g. Bonatti et al., 2005; Bowers et al., 2016; Cutting & Day, 1975) further undermines such arguments, as theories without phonemes cannot actually achieve the same coverage with less. Second, and more important, sceptics of phonemes have ignored the most important positive evidence for phonemes. In fact, phonemes were first hypothesized as units of lexical representation in linguistics in order to account for a variety of historical, morphological, syntactic, and semantic observations, and it is in this domain that the functional importance of phonemes is most clear (see, for example, the discussion of Baudouin de Courtenay in Anderson, 1985, p. 67: ‘[Baudouin de Courtenay took] the “phonemes” arrived at through the analysis of alternations to be the ultimate invariants of psychophonetic sound structure’). We consider the evidence from linguistics next.

## Part 3: Linguistic arguments for phonemes

The end goal of the listener is not merely to recognize individual morphemes or words but to understand the linguistic message overall, including recognizing the relations between morphemes inside the word and between words in phrases, sentences, and discourse. Consequently, language users must carry information forward from speech perception and word identification into subsequent morphological, syntactic, and semantic computations (Poeppel & Idsardi, 2011). It is this upstream computational aspect that makes phoneme-based representations central to linguistic theory, as operations at these higher levels require the ability to access a level of representation corresponding to single phoneme or a string of phonemes in order to carry out the relevant computations.

In what follows, we provide five arguments from various domains of linguistics that show that phonemes cannot be replaced with (demi-)syllables, contextual or positional variants of phonemes, or features.

### Subsyllabic and nonsyllabic words or morphemes

One form of evidence in support of phonemes comes from languages in which words can consist of a single consonant. For example, in Slovak there are four single-consonant prepositions, *k* ‘to’, *z* ‘from’, *s* ‘with’, and *v* ‘in’ (Hanulikova, McQueen & Mitterer, 2010; Rubach, 1993). Such phonological forms cannot be represented via syllables and call for segment-sized units (or smaller) in the lexicon and as perceptual access codes. In another language with single consonant words and words without any vowels, El Aissati, McQueen, and Cutler (2012) found that Tarifiyt Berber listeners showed equal abilities to spot words whether the remaining residue was a syllable or a single consonant.

The point above can be extended to a very wide range of languages if ‘words’ are replaced with ‘morphemes’. Morphemes are minimal pairings between a phonological form and a concept. Words are stand-alone morphemes (e.g. *table*) or combinations of morphemes (e.g. *government* consists of *govern* and -*ment*). Just like words, morphemes must be stored in the lexicon (moreover they are organizational units in the lexicon; see Marslen-Wilson, Tyler, Waksler, & Older, 1994, for psycholinguistic evidence on morphological organization of the lexicon). What is critical for our discussion is that morphemes are often below the size of a (demi-)syllable. For example, many common suffixes of English—the nominal plural morpheme /z/ (*dogs*), the verbal present tense third-person singular suffix /z/ (*he runs*), or the verbal past tense suffix /d/ (*played*)—are all single consonants. The important point is that it is not enough to recognize a word such as *books* or *played*, listeners also should be able to relate them to *book* or *play*. Without phonemes, these relations would be nontransparent and arbitrary, and these pairs would be no more similar than *cat* and *cap*, leading to a mounting load on the memory system.

In addition to words and morphemes that are smaller than a syllable, languages have root and affix morphemes that cannot be coherently represented via syllables. Consider a typical Semitic morphological family—such as Arabic *kitāb* ‘book’, *kutub* ‘books’, *kātib* ‘writer’, and *kataba* ‘he wrote’—that all relate to the concept of writing. On the phoneme view, the relation between these words can be represented elegantly by postulating that they share an underlying triconsonantal root *k*-*t*-*b* with vowel patterns reflecting different grammatical derivations. Such an account is supported by demonstrations that words like *kitāb* are decomposed into the consonantal root and a morphological pattern during lexical access (Arabic: Boudelaa & Marslen-Wilson, 2001, 2004; Hebrew: Frost, Deutsch, & Forster, 2000; Frost, Forster, & Deutsch, 1997). The (demi-)syllable view cannot encode bare consonantal roots because a sequence of consonants cannot be segmented into (demi-)syllables. Similarly, on the (allo)phone based view, the words would not share the same root as the consonant phones would differ depending on the vowel pattern. Again, this leads to an unsatisfactory outcome whereby a morphological relation between them is nontransparent.

An alternative could be proposed that morphologically related forms in Semitic languages are abstracted to an acoustic frame *k*-*t*-*b* that can vary the inner details (we thank Greg Hickok for pointing out this possibility). However, this view—as well as the demi-syllable and the (allo)phone based views—experience difficulty due to the existence of phonological processes in Hebrew that affect root consonants. First, the spirantisation process in Hebrew turns a stop into a fricative with the same place of articulation in certain contexts (primarily following vowels), for example, p → f, b → v, k → x. Accordingly, the root /k-t-b/ ‘write’ can be pronounced in several ways, depending on the position that the consonants occupy in the vocalised form (e.g. [yi-xtov] ‘he will write’ with /k-t-b/ here pronounced [x…t…v]). In addition, Hebrew has voicing assimilation for consonants in clusters; consequently, the first consonant of the root *k*-*t*-*b* can be pronounced in several ways, that is, [k, x, g] (modern Hebrew voicing assimilation does not create [ɣ]; Bolozky, 1997), as can the final consonant (i.e. [p, f, v, b]). So the acoustic template for the *k*-*t*-*b* root should be extended to {*k*, *x*, *g*}–{*t*, *d*}–{*p*, *f*, *v*, *b*}. But this template catches much more than just the desired root /k-t-b/ ‘write’ (e.g. the root /g-d-p/ ‘scorn, reproach’ also falls within it). Hence a template that corresponds to a common lexicosemantic representation cannot be formed solely on the basis of acoustic considerations.

### Recognizing morphemes and words in larger contexts

A strong rationale for postulating context-independent phonemes in linguistic theory is that they enable a parsimonious account of sound changes, alternation, and variation that takes place synchronically (i.e. at a given time) or diachronically (i.e. as a language changed through time). Synchronically, many pronunciation changes are associated with morphological derivation, as building larger forms often results into changes in how a constituent morpheme is realised phonetically. Next, we survey productive morphological processes in various languages to demonstrate that an adequate mapping between speech inputs and long-term memory requires phonemes as access codes.

#### Recognising morphemes in complex words

Across the world’s languages there are several ways in which morphemes combine to form words: suffixation, prefixation, infixation, and reduplication.

##### Suffixation and prefixation

Suffixation (adding a morpheme after the stem; e.g. *stamping*) and prefixation (adding a morpheme before the stem; e.g. *rewrite*) are the two most common morphological processes. Both processes ubiquitously lead to changes in the phonetic realisation of morpheme, in particular, to reassignment of phonemes to syllables (‘resyllabification’). For example, *stamp* [stæmp] combined with -*ing* [ɪŋ] yields *stamping* [stæm.pɪŋ], with /p/ resyllabified from the coda of the first syllable into the onset of the second. Now consider the task of recognizing *stamp* in *stamping*. If the speech perception system operates strictly on the basis of syllable-sized units, relating the input [stæm.pɪŋ] to the morpheme sequence {stæmp}{ɪŋ} during lexical access is an arbitrary associative process based on rote memory. That is, the relation between the syllable [stæm] and the morpheme {stæmp} would be no more similar than that between [ræm] ‘ram’ and {ræmp} ‘ramp’. On the phoneme view, on the other hand, the resyllabification of /p/ (so that the second syllable has an onset in line with a linguistic principle of ‘onset maximization’) does not affect the mapping process. Moreover, the /p/ moves *coherently* into the next syllable (i.e. the features comprising /p/ do not scatter between the two syllables, highlighting the point that the features are coordinated in time, the definition given for the phoneme above). Plentiful similar examples of resyllabification that yields misalignment between morpheme and syllable boundaries can be easily found for prefixation (e.g. in Russian the prefix /raz/ ‘extra’ combines with /o.det’/ ‘to dress’ to form /ra.zo.det’/ ‘overdress’) and across languages, emphasizing the universality of the phenomenon and need for a robust and general solution.

Resyllabification aside, suffixation and prefixation may induce other phonological changes including shifting stress away from the stem to a new location, leading to phonetic change inside the stem. For instance, adding the suffix -*ity* to the adjective *solid* [ˈsɒləd] with the stressed vowel [ɒ] yields *solidity* [səlɪdəti] with an unstressed [ə]. The pattern is widespread and extends to other suffixes (e.g. *compete* [k^h^əmp^h^it]—*competition* [k^h^ɒmpətɪʃən], *photograph* [fotəɡræf]—*photographer* [fət^h^ɒɡrəfə], atom [ærəm]—atomic [ət^h^ɒmɪk]). Note that there is no *solid* in *solidity* if phonological forms of words were represented via (allo)phones or (demi-)syllables. From the learner’s perspective, this means that knowing the word *solid* and its meaning is not the basis for deducing that *solidity* is about firmness or hardness. That this is clearly wrong has been known since Berko’s (1958) seminal demonstration of children’s remarkable ability to comprehend and generate novel morphological forms from just-learnt morphemes. An apparent solution for the (allo-)phone view is to store both allomorphs /sɒləd/ and /səlɪd/ in the lexicon. This would indeed allow recognizing the root *solid* in *solidity*, but at a high cost: How does the language user know which allomorph should combine with which suffix? What makes the combination of /sɒləd/ with /əti/ (i.e. *solidity* *[sɒlədəti]) illicit, or similarly /səlɪd/ with /-li/ (i.e. *solidly* *[səlɪdlɪ])? We also note that while perceptual proximity is an extremely useful dimension for contemplating and establishing morphological relatedness, it is insufficient to distinguish morphological versus nonmorphological relations among words (Stockall & Marantz, 2006). If *solidity* is related to *solid* on the basis of perceptual proximity, then what prevents relating *turnip* to *turn spinach* to *spin* on the same grounds? These problems do not arise with context-independent phonemes, which make it possible to encode that *solidity* contains *solid* as the stem: In both cases, the underlying vowel is /ɒ/ but a phonological rule reduces it to [ə] in an unstressed syllable.

##### Infixation

A more exotic type of word formation is infixation, whereby a morpheme is inserted within another morpheme. English employs this in a limited fashion in “expletive” infixation, as in *fan*-*bloody*-*tastic* or *in*-*fucking*-*credible*, whilst other languages use it productively. For example, in Tagalog the infinitive infix /-um-/ is added to /ʔa.bot/ ‘ability, reach’ to form /ʔu.ma.bot/ ‘to reach’, and to /pre.no/ ‘brakes’ to form /pru.me.no/ or /pum.re.no/ ‘to brake’ (Klein, 2005; Orgun & Sprouse, 1999). The infix, which often ends up straddling a syllable boundary, is added either after the first consonant or before the first vowel (and hence the variation in the form based on /preno/). But both the infix and some of the locations where it is inserted are definable only in terms of sequences of phonemes. Again, one could arbitrarily relate the two syllables /ʔa.bot/ with the three syllables /ʔu.ma.bot/, but given the frequency of infixation in Tagalog, that would imply a huge task for long-term memory. Furthermore, infixation continues to be productive, as seen by its application to borrowed words (e.g. /grad.wet/ ‘graduate’ yields /gru.mad.wet/ or /gum.rad.wet/ ‘to graduate’).

##### Reduplication

Another seemingly ‘exotic’ morphological process is reduplication, where some portion of a word is repeated to make a new form. Again, English makes limited use of this mechanisms, for example, in words such as *fancy*-*schmancy* (Nevins & Vaux, 2003). Other languages use reduplication much more productively, and in some cases the portion of the word that is repeated is not a syllabic or morphological unit. For example, in Mangarrayi (an indigenous Australian language), the plural of /waŋ.gij/ 'child' is /waŋ.g-aŋ.g-ij/ 'children' where the reduplicated VCC sequence -*aŋg*- does not constitute a whole syllable and spans a syllable boundary in the derived word (Raimy, 2000).

In sum, much of morphological derivation requires the ability to manipulate abstract segment-sized representations and cannot be adequately explained via (demi-)syllables or (allo-)phones. Phoneme-based encoding of lexical representations highlights the regularity in the lexicon and makes it more learnable.

#### Recognising words in phrases

Phonological changes do not only result from combining morphemes into words, they also result from combining words into phrases. Resyllabification across word boundaries is abundant across languages and can be exemplified via phonological phenomena of *enchainement* (‘chaining’) and *liaison* (‘linking’) in French (Schane, 1968; Tranel, 1987; Walker, 2001). In French, the combination of *nouvelle* /nu.vɛl/ ‘new’ and *amie* /a.mi/ ‘friend (feminine)’ yields the output [nu.vɛ.la.mi], with the final consonant of the adjective resyllabified into the initial syllable of the noun. Similarly, combining *amie* with *jeune* [ʒõe] ‘young’ or *petite* [pə.tit] ‘small’ results into [ʒœ.na.mi] or [pə.ti.ta.mi]. Liaison presents little difficulty to French listeners who can identify a word such as *amie* equally well whether it occurred in a canonical form or in a liaison context (Gaskell, Spinelli, & Meunier, 2002; Spinelli, McQueen, & Cutler, 2003). Yet if syllables were access codes to the lexicon, [nu.vɛl], [ʒœn], and [a.mi] could not be identified from the adjective-noun phrases due to misalignment between syllable and morpheme boundaries. An ostensible solution is to increase long-term storage (i.e. store multiple phonological forms [la.mi], [na.mi], [ta.mi], etc., alongside [a.mi] for the noun ‘friend’). The solution, however, does not work: It becomes impossible to know which combinations of phonological forms are (il)licit (i.e. what makes [nu.vɛl.na.mi] or [ʒœn.ta.mi] unacceptable; cf. a similar problem at the morpheme level in the section ‘Recognising morphemes in complex words’, above).

In sum, the process of combining morphemes into more complex words or words into phrases often leads to changes in the phonological form of morphemes or words. Such changes are regular and can be efficiently systematised only in a system that includes phonemes. Phoneme-based representations are required to encode resultant words and phrases compositionally and to enable a high degree of morphological transparency in the lexicon that is not achievable on the basis of (allo-)phones, (demi-)syllables, or features alone.

### Higher level/subsequent linguistic computation

Phoneme-sized representations are important beyond their role as access codes to the lexicon. Another critical function of phonemes relates to their role in signalling morphosyntactic relations in the sentence structure and for sentence interpretation. Some elements within a phrase or a sentence must agree (e.g. in *The boy is running*, the verb *is* agrees in number and person with the subject *the boy*). During language comprehension, the human parser verifies and uses such nonadjacent agreement relations to assign words to their positions in the syntactic structure (as shown, for example, by LAN and P600 event-related potentials; Coulson, King & Kutas, 1998; Osterhout & Holcomb, 1992; Osterhout & Mobley, 1995), and sometimes this step requires access to individual phonemes (even though by this point lexical access has been completed).

Consider the English possessive clitic ’*s*, represented by a single phoneme /z/. The naïve understanding of ’*s* is that it attaches to words to indicate possession or association, as in *the author*’*s book*, but in fact the clitic attaches not to a word but to an entire phrase. For instance, in *the king of France*’*s crown*, ’*s* is attached to and modifies not its local noun *France* but the entire phrase *the king of France* and its head noun *king* (hence, this phrase means that the crown belongs to the king, rather than to the state of France). Thus, there is a relation between a syntactic unit that consists of all and only the content of the subsyllabic, monosegmental clitic /z/ and the noun *king* at a distance. Another example comes from the English monophonemic plural suffix /z/. The final phoneme /z/ in *boys* must be recognized as the plural marker to establish patterns of agreement between the article and noun, as in *these* *three boys*, and between the subject and verbs, as in *The boys* *from New York* *are* *tired*.

To summarise, establishing relations between words and interpreting them as part of a larger syntactic and semantic structure requires verifying that morphosyntactic features (gender, number, case, etc.) properly agree. Ability to access these features requires phonemes; hence, phoneme-based representations must be visible to syntactic and semantic computations.

### Language games

Language games (Bagemihl, 1995; Sherzer, 1970; Vaux, 2011) are an elegant way of interrogating a language user’s linguistic knowledge. Similar to poetry, discussed in the next section, an aesthetic experience for the game player–listener is perceptual in nature (rather than being of articulatory nature) and informative for the discussion of units of speech perception. For example, the English Pig Latin game exhibits phoneme manipulation. In the most common version (or ‘dialect’) of Pig Latin players move the initial consonant cluster to the end and add [e] as in *blue* → [uble] (Barlow, 2001; S. Davis & Hammond, 1995). In another dialect of the game, only the first consonant is moved, as in *blue* → [lube]. The existence of the second dialect, particularly, reveals that a level of individual phoneme must be available to game players, and, in particular, that the players who are listening must be able to perceptually splice the received form [lube] to reconstruct the form for lexical access, [blu]. This is true even in syntactically complex forms, such as *Tom*’*s* (i.e. [tamz] → [amste]). In so doing, the listeners must ignore the difference between the tongue positions for /b/ in the game versus the real form of *blue* (before [e] in [lube] vs. before [l] in [blu]), or the devoicing of [z] in the game form of *Tom*’*s* (cf. [tamz] vs. [amste]), all while listening at normal speech rates.

Gil (1996) describes the Tagalog game Golagat, which reverses the whole phoneme sequence of the word (hence, the game’s name, the game form of [tagalog]). In the game forms, the corresponding phonemes are now in different syllable positions and contexts (e.g. the final coda /g/ of [ta.ga.log] is now a word-initial onset in [go.la.gat], whereas the initial /t/ becomes final). Speakers would have difficulty playing such a game if they only had access to allophones: Mapping the initial and final /g/ phone together requires an abstraction over them (i.e. a phoneme representation). Nor can the game be explained via syllables: The real and game forms do not share any syllables; hence, the language user would need to store a list of correspondences between real and game syllables only for the purposes of playing the game.

### Alliteration in poetry

Finally, phonemes are also necessary to describe poetic sound patterning, such as alliteration. We illustrate alliteration using three lines from the poem ‘Anglosaxon Street’, by the Canadian poet Earle Birney (Birney, 1975; for other examples of modern alliterative poetry, see Auden, 1947; Heaney, 2001):

go bleached beldames garnished in bargainbasements

festooned with shoppingbags farded, flatarched

….

like cutouts for kids clipped in two dimensions

As in traditional Germanic verse (Sievers, 1885), each line is divided into two half-lines, and the initial segments of one or more stressed syllable in each half-line must match. The alliterating phonemes are /b/, /f/, and /k/ in the first, second, and third lines, respectively, and as can be seen in these lines, clusters such as /bl/, /fl/, and /kl/ are allowed to alliterate with the single consonant onsets /b/, /f/, and /k/. Note also that in the third line, the /k/ s in *cutouts* and *kids* are pronounced differently (as a plain [k^γ^] and fronted [kʲ], respectively) because of the coarticulation in the context of the following vowel. This highlights the abstract nature of alliteration in that it ignores allophonic variation. The same abstraction away from allophonic variation is exhibited by 3-month-old children (Hayes & Slater, 2008) for alliterating sequences of /h/ in words and nonwords, such as *hig*, *hud*, *hat*, *hos*, *hem*, *hin*, *had*, *hut*, *hog*, *hug*, *ham*, in which there are various allophones of /h/ differing in tongue and lip positions, because the pronunciation of ‘h varies according to the vowel which follows it,…i.e. the h in these words is similar to the unvoiced vowels’ (Ward, 1929, pp. 149–50,). Alliteration improves memory for poetry (Atchley & Hare, 2013; Lea, Rapp, Elfenbein, Mitchel, & Romine, 2008), even alliteration of singleton consonants with the first consonant of a cluster (e.g. *g* with *gr* in alliterating lines such as ‘They let the ground keep the gold under the gravel, gone to the earth’; from Heaney, 2001).

Similar poetic patterns relying on individual phonemes can be found in verse composed in preliterate societies (e.g. *Beowulf* and other Germanic poetry; Foley, 1990; Heaney, 2001; Lord, 1960) or for young children, as in the poem “Singa Songa” (which uses both alliteration and rhyming) by the Canadian poet Dennis Lee (Lee, 1974, see below), indicating that their effect does not rely on literacy.

Singa songa sea

I’ve got you by the knee.

Singa songa sand

I’ve got you by the hand.

Singa songa snail

I’ve got you by the tail.

Again, in this poem, clusters must be unpacked so that *snail* alliterates with *singa*, *songa*, *sea*, and *sand*, and alliterative identity for /s/ must be adduced across the spectral changes in /s/ induced by the following vowels [a] or [i] (Yeni-Komshian & Soli, 1981). As noted above, the efficacy of these effects for drawing infants’ attention has been experimentally confirmed (e.g. infants prefer to listen to a sequence of alliterating words to that of nonalliterating words; Hayes & Slater, 2008; Jusczyk, Goodman, & Baumann, 1999). Preliterate children can also be better than adults at verbatim memory for rhyming texts (Király, Takács, Kaldy, & Blaser, 2017).

Likewise, Liu (1962) gives examples of alliteration in the Chinese poetry (known as *shuang sheng* ‘twin sounds’) from a prominent 8th-century poet Tu Fu (e.g. *Piao*-*po yiu pei chu*, *Chih* *chu tzǔ yi*-*ting* ‘Wandering abroad I still indulge in the cup, To and fro I pace in this post-pavilion’). Chinese is a nonalphabetic language, yet phonemes must be parsed out from the syllables in order to evaluate the lines of poetry for alliteration.

To conclude Part 3, a variety of linguistic data, including morphological derivation, language games, and poetry, demonstrate that linguistic generalisations require phonemes as units of representation. Access to phonemes is required beyond the immediate task of retrieving words from the lexicon but also to interpret syntactic and semantic relations between words in phrases or sentences. In language games and in poetry, phoneme-based regularities are widely used for producing an effect on the listener. Thus, phonemes must be access codes to the lexicon and a speech perception unit. Importantly, these linguistic observations extend to illiterates, again suggesting that phonemes are not a by-product of learning to read an alphabetic script.

## Part 4: From speech input to words via phonemes

In Fig. 2, we present our best current understanding of how the process of speech recognition works, given the discussion in the previous sections. In some ways, this is a return to ‘classic’ views on speech perception (Studdert-Kennedy, 1976) in that the model recognizes features, phones, phonemes, syllables, lexemes, and more. But rather than have a strictly pipelined approach (features first, then phones, then phonemes, etc.), we instead have the calculation and separation of information in parallel (i.e. demultiplexing the signal). In a similar vein, Pierrehumbert (2016) argues that listeners’ abilities to process novel forms and contexts requires a hybrid model which includes ‘an abstract level of representation…in which many phonetic details and contextual features are disregarded’ (p. 33). And another recent article, Fowler (2015) argues that ‘signatures of discrete, but temporally overlapping, segments are present in the signal’ (p. 125) and they strongly defend the notion of discrete phonetic segments, especially in articulation. But for the most part they do not address the specific questions of perceptual and computational abstraction raised above. Thus, we see Pierrehumbert (2006, 2016) and Fowler (2015) as consistent with the approach developed here, but placing different emphases on the various representations calculated during speech perception.

The incoming speech signal (at the very bottom of the figure) is filtered into critical bands in the peripheral auditory system and is represented in primary auditory cortex via a large number of spectrotemporal receptive fields (STRFs; David, Mesgarani, & Shamma, 2007; Mesgarani, David, Fritz & Shamma, 2008; Shamma, 2001, 2015). STRFs are somewhat like building blocks for spectrograms, and they can vary in time, frequency, and rate. Taken together, the STRFs provide a multiscale, multigrain, overcomplete analysis into neural representations for (at least) features (not separately shown), phones, phonemes (Mesgarani, Sivaram, Nemala, Elhilali, & Hermansky, 2009; Thomas, Patil, Ganapathy, Mesgarani, & Hermansky, 2010), and prosody in the middle layer of the figure, all of which are weakly coordinated in time. STRFs also can be used to classify speaker gender, identity (Coath, Brader, Fusi, & Denham, 2005), and emotional state (Wu, Falk, & Chan, 2011). We take this to indicate that the STRFs collectively perform ensemble decoding (Yildiz, Mesgarani, & Deneve, 2016); that is, they parcel out responsibility for various aspects of the incoming signal, effectively separating the speaker information from the message, and thereby normalizing the signal to extract phonemes. Various modules in the middle layer engage into cooperative lateral computation, symbolized here by the enclosing box, in much the same way the Kleinschmidt and Jaeger (2015) propose multiple joint inference of categories and indexical information. Representations from the middle layer then yield a lattice of possible word/morpheme continuations and segmentations which in turn activate conceptual representations.

Considering the specific example of input *camel* in Fig. 2, the initial aspirated [kʰ] is composed of the features {voiceless}, {stop}, and {velar}, which are detectable by STRFs and pass the activation up to both the phone [k^h^] and the phoneme /k/. (Note that although we use traditional feature names that refer to articulation, the features themselves are of double nature, i.e. grounded in both audition and articulation. Also, see the paragraph below for more information on phone and phoneme calculations.) Also, because of its aspiration, [kʰ] signals the beginning of a stress foot in English (shown as in the prosody box), a position that is also statistically predictive of the beginning of a word in English (Cutler & Carter, 1987; Cutler & Norris, 1988), and so would trigger an anchored search to word beginnings, as shown in the morpheme/word box. We hypothesize that the prosodic form of the word (mono- vs. bisyllabic, strong-weak vs. weak-strong syllable pair, etc.) is predicted from acoustic information very early in the word and accounts for the prosodic effects on word identification of *ham*/*hamster* type (Salverda, Dahan, & McQueen, 2003; see the section ‘Fine phonetic detail’, above). The existence of a (nonprimary) link to word and morpheme recognition through the prosody box should also account for listeners’ weak abilities to recognize sinewave speech (Remez, Rubin, Pisoni, & Carell, 1981) and temporal envelope speech (Drullman, 1995), both of which lack temporal fine structure cues. Cumulatively, the computations at the middle layer activate possible word/morpheme candidates, including, in this case, *camel*, *Camela*, and *Cam*’*ll* (= ‘Cam will’).

Returning to phone and phoneme computations in the middle of the diagram, sequences of phones are related to sequences of phonemes via the phonological regularities of the language. We emphasize sequences here because of the violations of linearity and biuniqueness (Chomsky, 1964), in which a property of a phone may signal information on a nearby phoneme. In our example, the receipt of the nasalized vowel [æ̃] in English signals both the (plain) phoneme /æ/ and makes a downstream prediction of a following nasal segment, shown in the diagram as a lattice of phoneme possibilities {/n/, /m/} (combining aspects of Church, 1987a,1987b; Lahiri & Reetz, 2002). This forward prediction is also the source of subphonemic detail effects on word identification (see discussion of Dahan et al., 2001, in the section ‘Fine phonetic detail’). Phone-to-phoneme lateral matching is restricted by language-specific phonological regularities and uses features as a metrics for comparison. Feature-based matching enables partial activation and access of words/morphemes on the basis of phones, as represented by a dotted line from phones to words/phonemes. However, it is an ordered phoneme-based representation which serves as a primary access code to the word/morpheme.

The primacy of phonemes (coordinated feature bundles) in our approach lies primarily in the preservation of the phoneme sequence in subsequent linguistic computations, such as the parsing of the English genitive clitic ’*s*, indicated in Fig. 2 by the persistence of phoneme representations at the word/morpheme level. But we also view phonemes as perceptually more primary (or immediate) than other approaches in that some STRFs are broadly tuned and can be activated by several different phones, thus forming equivalence-class (phoneme) detectors. Furthermore, the phoneme inventory at the middle layer in the system enables even higher order categories (e.g. categories that are activated by multiple distinct STRFs). Only a view with phonemes as perceptual units can account for the range of linguistic data reviewed in Part 3, as this view alone encodes systematic relations between phones and phonemes, so that representations for nonce forms can be constructed and relations between words can be computed. Likewise, only phonemes provide a perspicuous code in which different phonetic realisations of the same morpheme, such as [ˈsɒləd] (*solid*) and [səlɪd] (*solid*-*ity*), can be recognized, as the realizations contain the same phoneme string with different prosodic parsings. Yet we emphasize that in normal language comprehension, it is the cooperation of multiple modules from the mid layer and top-down influences from the lexical representations that underlies our ability to identify words so successfully (compare the relative difficulty of identifying phonemes reliably in nonce or foreign words).

The other proposals which come closest to ours in the recognition of the importance of phonemes to subsequent linguistic computations are those which posit phonemes following perception (Hickok, 2014; Morton & Long, 1976, though they differ in important respects; see Fig. 1). The fault we find with such systems is both empirical and conceptual. Hickok’s model in Fig. 1a that restricts phonemes to the stage of word-form encoding during speech production fails to account for any effects of phonemes in speech perception and comprehension, which includes most of the evidence discussed in Parts 2 and 3. In particular, we highlight that the linguistic evidence for phonemes in Part 3 cannot be attributed solely to the production system and clearly implicates the perceptual system. Indeed, language users are able to recognise morphemes and words that consist of a single consonant or Semitic roots that can be only represented at the segmental level, compute morphosyntactic relations in another speaker’s production, enjoy another person’s recital of poetry, and decipher the output of the player in a language game. Ability to show such performance requires ability to access phonemes within the perceptual system. Morton and Long’s (1976) model in Fig. 1b cannot account for phoneme effects during the initial perceptual stages, especially evident in phoneme monitoring studies on phonotactically illegal nonce words (Weber, 2002; see the section ‘Units of perception larger than phonemes: (Demi-)syllables’, above), which excludes any explanation based on lexical access or an inventory of valid syllables. Neither can the model account for the results of segment-based learning in artificial language learning tasks (Bonatti et al., 2005, also discussed in the abovementioned section). And as mentioned in Part 1, conceptually Morton and Long’s model leads to an odd outcome whereby any direct links between phones and phonemes are severed: If words are accessed via allophones and once retrieved just return a phoneme sequence for the word, then the correspondence between individual phones and phonemes is unknown. This seems to be untenable for multiple reasons; it is difficult to account for listeners’ awareness of distinct allophones such as [k^j^] in *key* and [k^ɣ^] in *cool* as belonging to the same phoneme category /k/, or for listeners’ difficulty in discriminating between different allophones of the same phoneme (e.g. the perceptual magnet effect; Kuhl, 1991). Finally, such models are just too profligate in their use of memory. Recognition of morphemes in the presence of resyllabification effects requires an enormous amount of tedious listing of forms that are predictable from the phoneme codes (as discussed in the section ‘Recognizing morphemes and words in larger contexts’, above).

Although phonemes are the core of the system that we propose, this is not to say that all language capacities are computed solely by the core phoneme system. Indeed, a range of psycholinguistic phenomena show that additional representations play a role in word perception and language processing more generally. For example, right hemisphere systems may encode the speech in quantitatively or qualitatively different ways in order to encode the emotional and various indexical features of language processing (Poeppel, 2003; Zatorre, 1997). Similarly, episodic memory within the hippocampus may contribute to various language-related tasks, including contributing to indexical effects on various processing tasks (see section ‘Indexical information’, above). However, we speculate that many of the indexical effects may turn out to be explained by the cooperative calculations among the representations in the middle of the diagram. That is, episodic memory for speakers may enhance phoneme recognition by ‘explaining away’ some of the speaker-specific acoustic idiosyncrasies; that is, performing another form of joint inference for nonprototypical values of acoustic parameters (such as excess VOT; see the section ‘Prototypicality effects across acoustic realisations’, above), akin to Kleinschmidt and Jaeger (2015)

However our goal here is not to detail how the right hemisphere, episodic memory, and all the various sublexical phonological representations interact in order to support speech perception and comprehension. Rather, our goal is to argue that phonemes are necessary and serve as access codes to lexemes. On our view, any attempt to discard phonemes for alternative sublexical representations will fail, and the main reason why so many theorists have attempted to replace phonemes with various alternative sublexical representations is that they have not considered the core reasons why phonemes were introduced in the first place, namely, various linguistic phenomena concerning how complex word forms and syntactic processes are accomplished, which require access to subsyllabic feature bundles coordinated in time.

## Footnotes

1 Proximity of the concept of the molecule in articulatory phonology to phonemes has been explicitly asserted by one of the proponents of the theory, Carol Fowler: “I am convinced by the success of alphabetic writing systems, and the approximately segmental character of a substantial subset of sublexical speech errors that the particles are not far from conventional segments” (Fowler, 2015, p. 40).

2 An anonymous reviewer notes that some phonological theories, such as optimality theory (OT), do not make use of phonemes as described above and instead derive morphophonological regularities in the language via an interaction between equivalence sets of underlying and surface forms and constraints on them (Prince & Smolensky, 2008; see also Baković, 2014, for a brief discussion). Although the exact mechanism of representing phonological forms of words in the long-term memory using the equivalence classes is not fully clear to us, we point out this alternative. In our view, the OT equivalence classes require abstraction over segments and thus are comparable to a phoneme (at least to the degree that makes the OT and phoneme-based approaches fall on the same side of the debate vis-a-vis claims rejecting abstract segment-sized units in the speech perception literature discussed in Part 2).

3 Pierrehumbert (2002, 2003; see the section ‘Contextual variants of phonemes: Effects of phoneme variability due to neighbouring segments’) uses a similar logic to reject phonemes in favour of position-specific variants of phonemes as sublexical units in speech perception due to them being more invariant in acoustic terms.

4 Although Massaro’s claims are formulated in terms of syllables, they should be more appropriately called ‘demi-syllables’. This is because in Massaro’s approach CVC syllables are considered to be segmented into CV and VC units (*V* stands for a vowel, *C* stands for one or more consonant; Massaro & Oden, 1980). Hence, Massaro’s perceptual units are V, CV, and VC demi-syllables. We use the notation (demi-)syllables to refer to such units. Massaro’s rationale for representing CVC syllables as a combination of two units (i.e. the CV and VC demi-syllables) is due to the necessity for the unit to be no longer that 250 ms (whereas CVC sequences can be longer). Note however that this approach requires explicit listing of which VC’s can legitimately follow each CV demisyllable in order to prevent overgeneration of illicit CVC syllables in English such as /beuk/ (combined from the demi-syllables /be/ and /uk/).

5 It is noted that in principle the outcome may be obtainable via multisyllable templates (Greg Hickok, personal communication); however, to date this position has not been elaborated in sufficient detail in the published literature, hence its tenability will not be discussed further.

6 We thank an anonymous reviewer for bringing this point to our attention. A single exception to phoneme preservation is when /r/ in the input is substituted for /l/ in the fused form, e.g. *pay* – *ray* pair yielding *play*, which is attributed to an independently known fact of instability of /r/ in perception (Cutting, 1975).

7 The issue of the size of the sublexical representations in speech overlaps with the issue of how units are bound to positions within a syllable or word discussed in the section ‘Positional variants of phonemes: Variability across syllable or word position’ (i.e. whether or not segments are invariant across syllable/word positions).

8 In this section we discuss the case of segmental variation that is restricted to the critical segment being in the same position within a word or syllable but surrounded by different segments. In the section ‘Positional variants of phonemes: Variability across syllable or word position’, we consider phoneme variability due to varying position within a word or syllable.
